# Successful Use of Negative Pressure Wound Therapy for Abdominal Wall Necrosis Caused by a Perforated Ascending Colon Using the ABThera System

**DOI:** 10.1155/2020/8833566

**Published:** 2020-07-21

**Authors:** Kouki Imaoka, Takuya Yano, Yasuhiro Choda, Ko Oshita, Yuma Tani, Tetsushi Kubota, Michihiro Ishida, Daisuke Satoh, Masanori Yoshimitsu, Kanyu Nakano, Masao Harano, Hiroyoshi Matsukawa, Hitoshi Idani, Shigehiro Shiozaki, Masazumi Okajima

**Affiliations:** ^1^Department of Surgery, Hiroshima City Hiroshima Citizens Hospital, 7-33 Motomachi, Naka-ku, Hiroshima 730-8518, Japan; ^2^Department of Gastroenterological and Transplant Surgery, Applied Life Sciences, Institute of Biomedical & Health Sciences, Hiroshima University, 1-2-3 Kasumi, Minami-ku, Hiroshima 734-8551, Japan

## Abstract

**Background:**

The practice of leaving the abdomen open after an emergency laparotomy has gained increasing popularity recently. Negative pressure wound therapy (NPWT) is known as an effective technique in the management of an open abdomen (OA). A new device, the ABThera™ Open Abdomen Negative Pressure Therapy System (KCI USA, San Antonio, TX, USA), was specifically designed to achieve a temporary abdominal closure (TAC) in the management of an OA. This study was aimed at presenting a successful experience of treating a case of abdominal wall necrosis caused by a perforated ascending colon using the ABThera System. *Case Presentation*. A 66-year-old man was admitted to our hospital with complaints of severe pain in the abdomen. On admission, abdominal contrast-enhanced computed tomography (CT) showed fluid collection, an air pocket in the subcutaneous fat layer of the abdominal wall, and edematous changes in the adipose tissue in the peritoneum and abdominal wall. Based on a diagnosis of peritonitis resulting from a perforated ascending colon, emergency surgery was performed. A right hemicolectomy, ileostomy construction, and debridement of the necrotic tissues were performed. However, necrotizing fasciitis rapidly spread; therefore, more necrotic tissue was debrided in a second operation. The abdominal wall defect was left open, and the ABThera System was used in the management of the OA; this device promoted wound healing. A reduction was observed in the size of the open wound with visible granulation tissue. The defect was finally covered with a mesh split-thickness skin graft and anterolateral thigh flap.

**Conclusions:**

In the management of a case of a massive wound with infection, it can be of great benefit to treat the wound with NPWT initially to decrease its size. The ABThera System could facilitate early and safe management of an OA by surgeons.

## 1. Introduction

The practice of leaving the abdomen open after an emergency laparotomy has gained increasing popularity recently. Patients with an open abdomen (OA) require temporary abdominal closure (TAC) to allow for a period of stabilization prior to definitive closure. There are multiple techniques to facilitate TAC in the management of an OA [1–5]. Among these techniques, negative pressure wound therapy (NPWT) is the most effective approach [6]. However, there is no device for the performance of NPWT in Japan that is specifically designed for the management of patients with an OA. A new device for TAC, the ABThera™ Open Abdomen Negative Pressure Therapy System (ABThera; KCI USA, San Antonio, TX, USA) was approved in March 2019 for use in Japan. This device is specifically designed for the management of OA. We present a successful experience in the treatment of abdominal wall necrosis caused by a perforated ascending colon using the ABThera System.

## 2. Case Presentation

**Figure 1:**
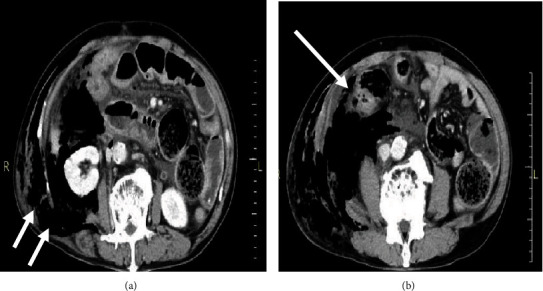
Radiological findings: (a) abdominal contrast-enhanced computed tomography (CT) scan showing fluid collection, an air pocket in the subcutaneous fat layer of the abdominal wall, and edematous changes in the intraperitoneal and abdominal wall adipose tissue (arrow); (b) inflammatory changes are observed around the ascending colon (large arrow).

**Figure 2:**
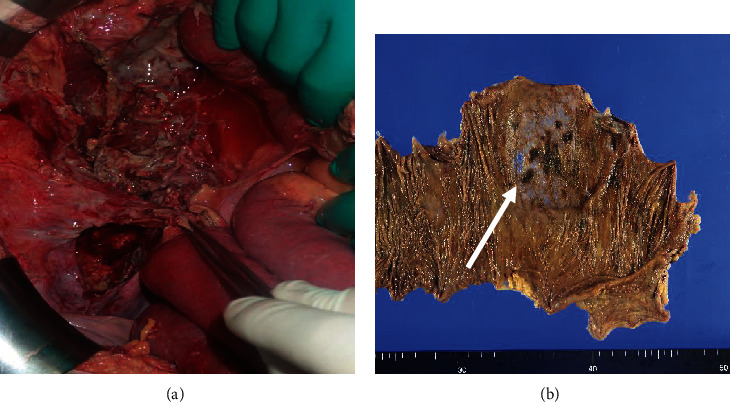
The initial surgical and histological findings: (a) the surgical findings revealing extensive necrosis of the retroperitoneal fat around the ascending colon where the inflammation is severe; (b) right hemicolectomy specimen. The pathological findings of the resected specimen reveal peritonitis, ischemic changes, and thinning of the wall of the ascending colon with no malignancy (arrow).

A 66-year-old man was admitted to our hospital with complaints of severe pain in the abdomen, which persisted for over 48 hours. The medical history included a splenectomy after trauma and a posttotal gastrectomy for gastric cancer. On admission, tenderness and involuntary guarding were observed in all quadrants of the abdomen. Additionally, the patient developed fever (temperature of 39°C) and was hemodynamically unstable with a blood pressure of 90/50 mm Hg and a heart rate of 120 bpm. On physical examination, skin discoloration was observed around the right side of the abdomen with tense edema. Laboratory tests revealed a C-reactive protein level of 18.0 mg/dL (range, 0-0.30 mg/dL), white blood cell count of 2700/mm^3^ (range, 3300-8600/mm^3^), and an elevated serum creatine kinase level of 1977 U/L. Abdominal contrast-enhanced computed tomography (CT) showed fluid collection, an air pocket in the subcutaneous fat layer of the abdominal wall, and edematous changes in the adipose tissue in the peritoneum and abdominal wall (Figure 1(a)). Inflammatory changes were observed around the ascending colon (Figure 1(b)). Based on a diagnosis of peritonitis resulting from a perforated ascending colon, an emergency operation was performed. Extensive necrosis of the retroperitoneal fat was observed around the ascending colon where the inflammation was severe. In addition, necrotizing fasciitis was suspected in the abdomen due to an infection caused by the perforated ascending colon. The patient was immediately managed with an intravenous administration of a broad-spectrum antibiotic (meropenem at 1.5 g/day). A right hemicolectomy, ileostomy construction, and debridement of necrotic tissues were performed (Figure 2(a)). The stump of the ascending colon was closed without colostomy. The pathological findings of the resected specimen revealed peritonitis and ischemic changes, and the ascending colon was noted to be very thin; however, no malignancy was detected. (Figure 2(b)).

**Figure 3:**
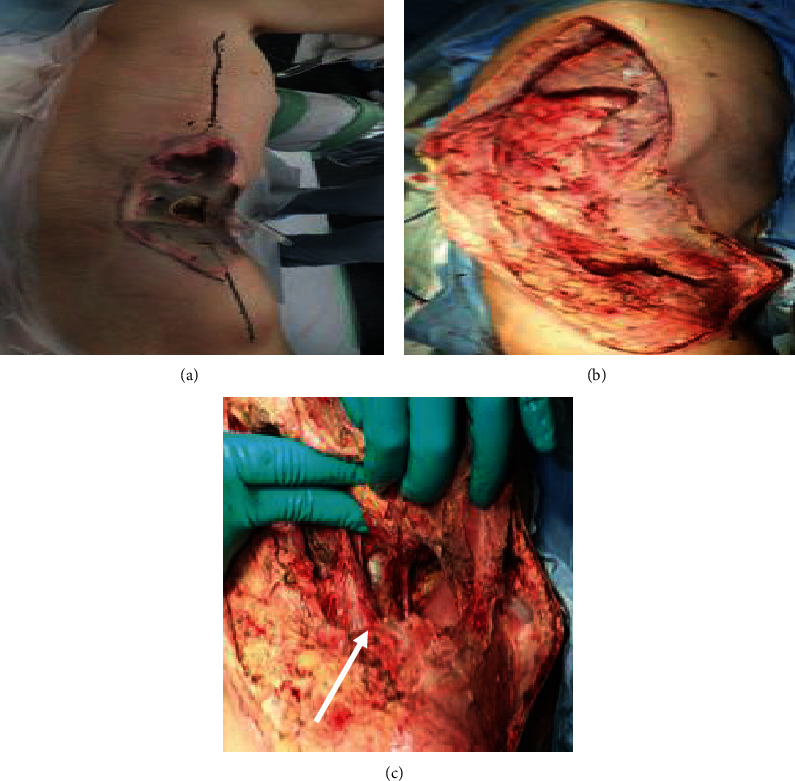
The surgical findings at the second operation. (a) Two days after the initial operation, skin discoloration is observed around the right side of the abdomen. (b) Necrotic tissues were debrided from the level of the right abdominal wall to the affected skin and subcutaneous tissue on the right side of the chest wall. (c) The abdominal wall defect. The intestine, inferior vena cava, and right kidney are exposed (arrow).

**Figure 4:**
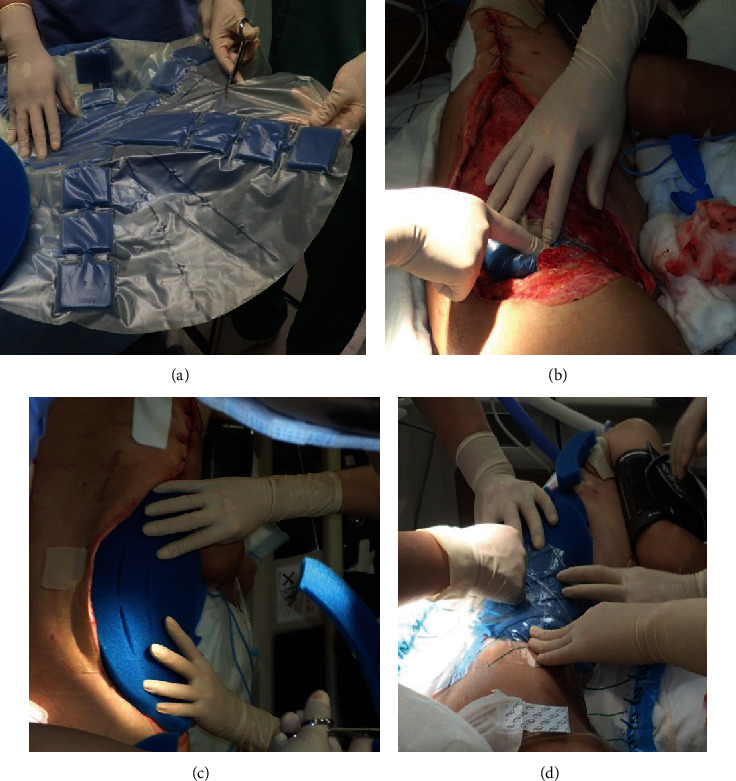
The use of ABThera. (a) The ABThera System has a different inner layer design and is composed of a polyurethane film-covered central foam structure with six arms. (b) Installation of the ABThera System involved placing the fenestrated plastic dressing with the incorporated polyurethane sponge over the viscera to protect the abdominal contents. (c) A GranuFoam sponge was subsequently placed over the plastic sheet. (d) The abdomen was sealed with an adherent plastic sheet to the skin, and the ABThera System was set to –125 mmHg continuous suction.

**Figure 5:**
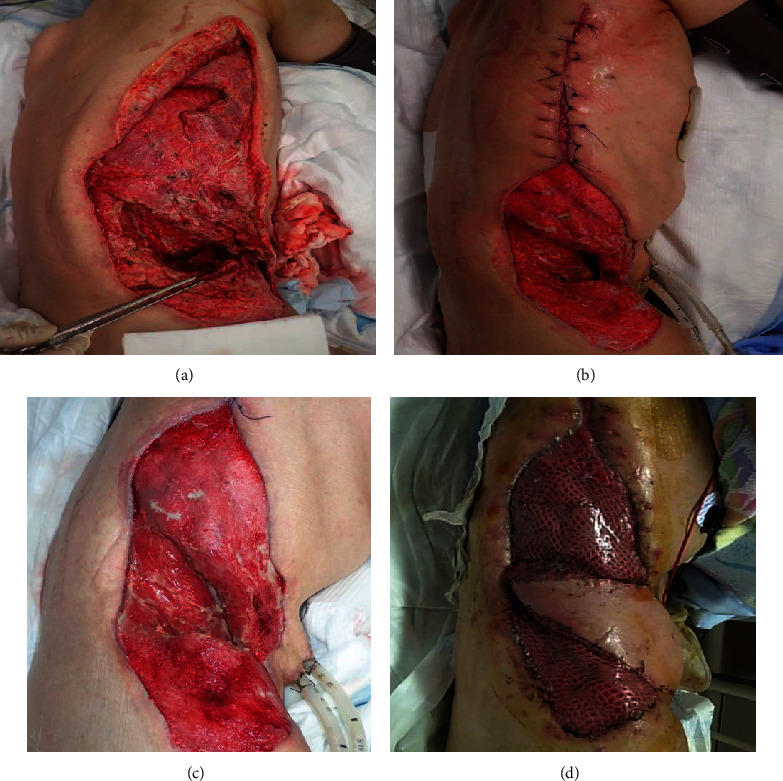
Photographs of the wound. The size of the open wound with visible granulation tissue was reduced using the ABThera System. (a) Postoperative day (POD) 10. (b) POD 25. (c) POD 30. (d) The abdominal wall defect was covered by a mesh split-thickness skin graft and anterolateral thigh flap.

**Figure 6:**
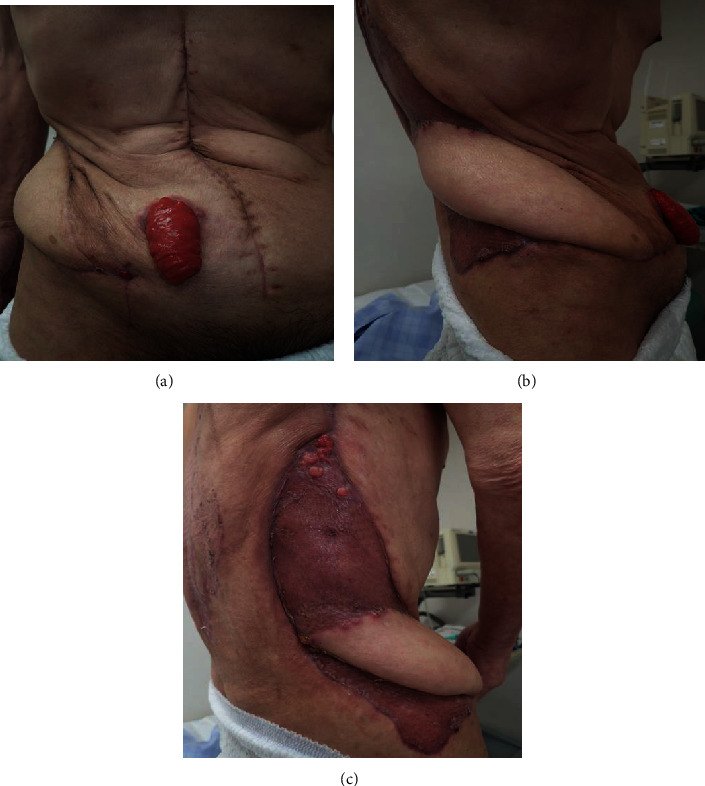
The final appearance of the abdomen: (a) frontal view; (b) right lateral view; (c) dorsal side view.

Two days following the initial operation, the skin discoloration around the right side of the abdomen reappeared, which indicated widespread necrosis of the abdominal wall fascia (Figure 3(a)). The follow-up CT showed necrotizing fasciitis spreading up to the right lateral chest wall, along the right lateral abdominal wall, and extending to the right groin. A second operation was performed, and the necrotic tissue was debrided from the level of the right abdominal wall to the damaged skin and subcutaneous tissue on the right side of the chest wall (Figure 3(b)). This resulted in a significant abdominal wall defect. The intestine, inferior vena cava, and right kidney were exposed (Figure 3(c)). A closure of the wound defect was planned using the ABThera™ System to remove exudate and promote wound healing (Figures 4(a)–4(d)). Under local anesthesia, repeated surgical debridement was performed, and the NPWT dressing was changed 3 times a week for 4 weeks. Following the application of NPWT set at 125 mmHg of continuous negative pressure, extensive debridement and NPWT successfully contributed to the wound bed healing. The size of the open wound with visible granulation tissue significantly reduced with no complications such as an enteric fistula (Figures 5(a)–5(c)). After 4 weeks of NPWT, the wound beds were sufficiently developed for plastic surgery. Finally, the defect in the abdominal wall was covered by a mesh split-thickness skin graft and an anterolateral thigh flap (Figure 5(d)). The patient was discharged 63 days after admission and was regularly followed up at the outpatient department for 6 months without complications (Figure 6).

## 3. Discussion

The term “open abdomen” (OA) refers to a surgically created defect in the abdominal wall that exposes the abdominal viscera. Leaving the abdomen open after an emergency laparotomy (for example, after damage control surgery (DCS) or abdominal compartment syndrome (ACS)) has gained increasing popularity recently. The benefits of managing patients with OA include prevention of intra-abdominal hypertension (IAH), ACS, early identification of intra-abdominal complications (e.g., bowel ischemia), and ease of reoperation. However, patients with OA do require temporary abdominal closure (TAC) to allow for a period of optimization prior to definitive closure. There are multiple techniques associated with the management of OA that can be used to facilitate TAC, such as loose packing of the abdominal cavity [1], use of towel clips [2], placement of mesh materials [3], use of polyvinyl bags [4], or even use of textile and zipper-like devices [5]. Until 2016, there was no consensus as to which treatment option was superior, although various studies indicated that negative pressure wound therapy (NPWT) and its variants were the most effective approach, yielding the best results and reducing associated complications [6]. In most centers, the use of abdominal NPWT has become a standard treatment in OA patients. The application of negative pressure to a wound increases dermal perfusion and stimulates the formation of granulation tissue, thus accelerating wound healing and decreasing bacterial colonization; this is because it reduces tissue edema and interstitial tissue fluid [7, 8]. The efficiency of NPWT has been proven, and it is currently used in the treatment of trauma-induced soft tissue defects, necrotizing fasciitis (NF), suppurative and extravasation injuries, and burn wounds and in promoting skin graft fixation [9, 10]. Batacchi et al. observed that the time to wound closure in a group of patients treated with NPWT was shorter than that in patients for whom a Bogota bag was used (4.4 vs. 6.6 days, *p* = 0.025). In addition, the length of median Intensive Care Unit and hospital stay was shorter in NPWT patients than in the patients treated with the Bogota bag (13.3 and 6 days, and 28.5 and 21 days, respectively) in the prospective study [11]. The advantage of reducing ICU length of stay using NPWT was similarly confirmed by a recent systematic review [12].

The ABThera™ System was approved in March 2019 for use in Japan. It assists surgeons in promptly assuming responsibility in the management of OA and to achieve primary fascial closure. The ABThera System is designed to remove fluid and reduce edema, provide medial tension (which helps to minimize fascial retraction and loss of domain), facilitate the isolation of the viscera and abdominal contents from the external environment, and ensure separation between the abdominal wall and the viscera, thereby protecting the abdominal contents.

Evidenced by the values obtained for the parameters of comparison (pressure delivery, pressure distribution, fluid removal, and performance consistency), Delgado and Sammons reported that the ABThera System was a superior method of TAC compared to Barker's vacuum packing technique (BVPT) and the V.A.C. Abdominal Dressing System [12]. The result of this in vitro study was supported by data on clinical outcomes. Cheatham et al. reported the 30-day all-cause mortality as 14% and 30% for ABThera and BVPT, respectively (*p* = 0.01). Furthermore, multivariate logistic regression analysis demonstrated that patients treated with the ABThera System were significantly more likely to survive than those treated with BVPT (odds ratio 3.17 (95% confidence interval 1.22-8.26); *p* = 0.02) after controlling for age, severity of illness, and cumulative fluid administration [13]. In addition, the reverse tissue expansion effect of negative pressure facilitates the approximation of the skin and fascia. NPWT can equally retract the abdominal walls toward the midline and allow approximation of the wound edges. Cheatham et al. observed that the median number of days to primary fascial closure (PFC) was 9 and 12 days for the ABThera System and BVPT, respectively (*p* = 0.12). The 30-day PFC rates were 69% and 51% for the ABThera System and BVPT, respectively (*p* = 0.03) [13]. Improved PFC rates have been demonstrated to correlate with significant increases in patient survival and decreases in hospital charges [13–15]. Moreover, the ABThera System is more convenient for dressing and preventing infections because replacement of the wound dressing is only needed 2 to 3 times a week.

There have been concerns regarding the use of NPWT in OA in terms of fistula development [16, 17]. However, in a published meta-analysis, the V.A.C. technique was not associated with a higher risk of fistula formation [18], and Cheatham et al. reported no observed difference in the incidence of critical complications such as the development of ACS or an intestinal fistula during TAC therapy between ABThera and BVPT [13].

NF is an uncommon condition characterized by a necrotic infection that rapidly diffuses along the fascial plane and progresses to systemic sepsis. The male-to-female ratio is 3 : 1, mainly due to the higher incidence of Fournier's gangrene in males [19]. The occurrence of NF of the abdominal wall due to a perforated colon is extremely rare. Thorough and immediate debridement remains the cornerstone treatment for NF, and the resulting defects can be massive. The use of NPWT in NF therapy started in 1999 and has been very effective for the management of massive wounds [20]. In case of a massive wound, there can be significant benefit in initially treating the wound with NPWT to decrease its size. A successful experience has been reported treating abdominal wall necrosis caused by a perforated ascending colon using the ABThera System. However, a prospective comparative study is required to confirm the usefulness and safety of the ABThera System in patients with abdominal wall necrosis due to infection.

## 4. Conclusion

The study findings can be availed by traumatologists, surgeons, and related clinicians in the course of clinical practice to effectively manage extensive infected wounds, thereby maximizing the desirable clinical outcome in affected patients.

A successful experience has been reported treating a patient with abdominal wall necrosis caused by a perforated ascending colon using the ABThera™ Open Abdominal Negative Pressure Therapy System. The limitation of this study was that it was difficult to achieve primary fascial closure due to the extensive abdominal wall defect; however, the ABThera System facilitated a reduction in the wound size. In managing a case of a massive infected wound, initial treatment of the wound with NPWT can be of significant benefit.
